# Preliminary analysis of immune responses in patients enrolled in a Phase II trial of Cyclophosphamide with Allogenic DRibble Vaccine Alone (DPV-001) or with GM-CSF or Imiquimod for adjuvant treatment of Stage IIIA or IIIB NSCLC

**DOI:** 10.1186/2051-1426-2-S3-P249

**Published:** 2014-11-06

**Authors:** Traci Hilton, Rachel Sanborn, Brian Boulmay, Rui Li, Bradley Spieler, Kyle Happel, Christopher Paustian, Tarsem Moudgil, Christopher Dubay, Brenda Fisher, Eileen Mederos, Augusto Ochoa, Walter J Urba, Hong-Ming Hu, Bernard Fox

**Affiliations:** 1 UbiVac, USA; 2 Robert W. Franz Cancer Research Center, Earle A. Chiles Research Institute, Providence Cancer Center, Portland, OR, USA; 3 Stanley S. Scott Cancer Center, School of Medicine, LSUHSC, New Orleans, LA, USA

## Background

Tumor-derived autophagosomes, DRibbles, are novel cancer vaccines that have been shown to be effective in preclinical models of established tumors. We hypothesize that DRibbles' efficacy stems from their ability to present stabilized tumor-derived short-lived proteins (SLiPs) and defective ribosomal products (DRiPs) that are normally not processed and presented by professional antigen presenting cells (APCs). These SLiPs and DRiPs represent a potential pool of tumor antigens against which the host is not tolerant. The DPV-001 vaccine packages at least 40 putative cancer antigens, including twelve antigens from the NCI's list of prioritized antigens, multiple DAMPs and agonist activity for TLR 2, 3, 4, 7 and 9 into stable double membrane microvesicles. These microvesicles have surface f-actin and spectrin that target DRibbles to CLEC9A+ APCs, the most effective APC at cross-priming immunity.

## Methods and Results

Patients are vaccinated at 3-week intervals with a total of 7 doses. PBMCs and serum are collected at baseline and at each vaccination. Immune monitoring panels are run on peripheral whole blood to evaluate lymphocyte populations and their activation. PBMCs from the baseline visit and week 12 are treated with DRibbles (vaccine and control) to measure vaccine specific cytokine production. Patient serum from the baseline visit and week 12 is analyzed for antibody response to >9000 human proteins with ProtoArrays. We have identified multiple induced and increased antibody responses, after vaccination (n = 2 patients). UbiLT3 and UbiLT6 mutations were compared to lung adenocarcinoma (LUAD) patient mutations in the TCGA database (http://www.cbioportal.org/public-portal/data_sets.jsp) to identify mutations shared with the DPV-001 vaccine (UbiLT3 average 78.6; UbiLT6 average 73.9 shared SNPs, excluding dbSNP (common) variants). http://www.syfpeithi.de/bin/MHCServer.dll/EpitopePrediction.htm was used to find the ligation strength of reference and mutated sequences on HLA types in order to predict potential T cell epitopes.

## Conclusions

The DPV-001 vaccine provides a source of broad-spectrum relevant antigens. Preliminary analyses of patients receiving the DPV-001 vaccine show effects on T cells and increased humoral antibody responses at 12 or 13 weeks. The DPV-001 vaccine contains mutations shared with NSCLC patients in the TCGA database, including mutations with increased immunogenicity that represent potential therapeutic targets.

## Clinical trial identifier

NCT01909752

## Support

R44 CA121612-02A1

